# Autochthonous *Arthrospira platensis* Gomont Driven Nickel (Ni) Phycoremediation from Cooking Oil Industrial Effluent

**DOI:** 10.3390/molecules27165353

**Published:** 2022-08-22

**Authors:** Isha Shakoor, Aisha Nazir, Sonal Chaudhry, Qurat-ul-Ain, Firdaus-e-Bareen, Sergio C. Capareda

**Affiliations:** 1 Environmental Biotechnology Laboratory (F4), Institute of Botany, University of the Punjab, Lahore 54590, Pakistan; 2 Department of Botany, Lahore College for Women University, Lahore 54000, Pakistan; 3 Institute of Molecular Biology and Biotechnology, Faculty of Sciences, University of Lahore, Defence Road, Lahore 54000, Pakistan; 4 Department of Biological and Agricultural Engineering, Agriculture & Life Sciences, Texas A & M University, College Station, TX 77843, USA

**Keywords:** algal biomass, biosorption, phycoremediation, nickel, oil industrial effluent

## Abstract

Nickel (Ni) leftovers arise from both catalyst application interventions and Ni alloy piping of the cooking oil industry (COI) being wasted as pollutants of freshwater bodies via discharged effluent. The current study assessed one of the indigenously feasible Ni removal systems comprising autochthonous *Arthrospira platensis* Gomont (AP)-driven Ni phycoremediation cells (NPCs). After screening AP for hyperaccumulation in the Ni spiked solution, AP was transferred to the NPCs. Propagation of the AP inoculum was proportionate to the pollution load drop of COI with 22.97 and 55.07% drops in the biochemical (BOD) and chemical oxygen demand (COD), respectively. With the 0.11 bioconcentration factor, there was an uptake of 14.24 g mineral with 16.22% Ni removal and a 36.35 desorption ratio. The experimental data closely fitted with the Langmuir and Freundlich isotherms, respectively. The study concluded that *A. platensis* could be taken for treatment of Ni-loaded industrial effluents at the microcosmic level.

## 1. Introduction

Global expansion at the social and economic level has led to an increase in industrial processes, municipal discharge, mining activities and agricultural runoff resulting in water pollution [1,2]. Nickel pollution has increased around the globe due to the indiscriminate and unregulated release of industrial wastewater being one of the greatest threats to environmental sustainability, biodiversity, microfauna and soil properties [3]. Nickel is among the 23 metals along with Cd (Cadmium) and Pb (Lead), characterized as major pollutants of our environment and was titled “allergen of the year” in 2008 [4]. The maximum permissible limit for Ni (Nickel) by WHO (World Health Organization), USEPA (United States Environmental Protection Agency) and NSDWQPak (National Standards for Drinking Water Quality-Pakistan) in drinking water is 0.07 mg L^−1^, 0.5 mg L^−1^ and 0.02 mg L^−1^ respectively.

Punjab Board of Investment and Trade has declared Gujranwala to be the third largest industrial city in Pakistan, producing 71 million gallons of wastewater annually, which drains into a main stream line of Saim Drain and eventually empties into River Chenab [5]. The oil and ghee sector produces an average of 12,600 m^3^ of effluent per day [6]. This wastewater contains organic and inorganic contaminants, such as mineral acids and hazardous metals, which can deplete oxygen and cause diseases. The processed wastewater is rich in oil, grease, Chemical Oxygen Demand (COD), Biological Oxygen Demand (BOD), Total Dissolved Solids (TDS), Total Suspended Solids (TSS), Sulphates (S^2−^), Chlorides (Cl^1−^), Phosphorous (P^3−^) and Nickel (Ni^2+^) whereas the auxiliary wastewater has a high temperature and high Volatile Organic Compounds (VOCs) content. Wastewater consists of 95% water and 5% solids, out of which 50% are suspended ones, including 0.7% of oil. The concentrations of oil and grease in untreated domestic wastewater are between 50 and 100 mg L^−1^ [7]. Nickel is used as a catalyst during the process of hydrogenation to prevent the decomposition and oxidation of oil [8]. The ghee is then filtered to remove the catalyst and washed thrice with boiled water during the post-neutralization process [9]. 

The physiochemical processes for the removal of carcinogenic metals are either expensive or ineffective for the treatment of high metal concentrations. For biosorption of Ni, a variety of bacteria, fungi, algae, yeast and plants have been investigated. As heavy metals such as Cd^2+^, Pb^2+^, Cu^+2^, Cr^+3, +6^, Zn^+2^, Ni^+2, +3, +4, 0, −1^, Hg^2+^, Ar^+^, Ag^+1^, Fe^2+^,^3+^ etc. persist in the environment, microalga have the potential to absorb and accumulate these substances [10]. Microalgae may effectively treat agricultural, industrial dairy, piggery and municipal wastewater as they utilize the minerals, such as Nitrates, Phosphates and CO_2_ present in them, ultimately leading to a stable ecosystem [11]. They are economic, ecofriendly and cost effective with high regeneration and potential for survival and adaptation in unfavorable conditions of salinity, heat, cold, light, osmotic pressure, oxygen depletion and metal stress [12].

*Arthrospira platensis* Gomont, a Gram-negative microalgae sold under the trade name *Spirulina platensis* (Voronich.) Kom and Lund (1990) are the only microalgae cultivated commercially for supplements, probiotics and chemicals in bulk quantities. It thrives in freshwater and extremoalkalophilic and halophilic environments. In outdoor and open ponds, the monoculture of *Arthrospira* has been successfully produced because high alkalinity favors its production and acts as a barrier to extraneous contamination. It shows growth at pH 9.0–10.0, high bicarbonate concentration and a range of temperatures between 24–42 °C [13]. The tolerance and detoxification capability of *Arthrospira* is valuable for the rapid removal of multimetals simultaneously from industrial wastewater at a low cost [14]. The use of autochthonous and indigenous microbes isolated from heavy metal-polluted environments seems feasible. 

The objectives of the current study were to improve knowledge of biological and cost-effective methods of wastewater treatment systems in Pakistan. The present study focuses on: (i) isolation, identification and culturing autochthonous microalgae Arthrospira collected from contaminated Saim Drain, Gujranwala; (ii) investigation of physico-chemical and biological parameters of wastewater samples of Prime Oil and Ghee industry, Gujranwala; and (iii) assessment of remediation potential of *Arthrospira platensis* Gomont for treatment of nickel-contaminated wastewater under laboratory and natural conditions.

## 2. Results

### 2.1. Biomass Assessment of Arthrospira platensis and Physico-Chemical Characterization of Wastewater

The microalgal samples were observed as bright green pea color fuzzy masses floating in discrete patches along the embankment of the Saim Drain. The microalgae were identified as *Microcyctis*, *Oscillatoria* and *Arthrospira* after microscopy of centrifuged samples on the basis of morphological and phenotypic characteristics. *Microcystis* and *Oscillatoria,* which produce toxic microcystins and nodularins, were excluded from the study and *Arthrospira* was selected as the experimental strain due to its efficacy in nutrient removal from wastewater, as documented in the literature. *Arthrospira* were cultured in BG-11 medium over a span of 21 days. Their biomass analyses are presented in Table 1.

The color of wastewater from the Prime Oil and Ghee industries was characterized as greenish blue to bluish green according to the Floral-Ule index corresponding to a 30.436° Hue color angel [15] with a threshold odor number (TON) of 4. The turbidity in terms of secchi disk depth was 6.5 cm.

The pH, temperature, TDS, TSS, Cl, S, Ca, Mg, hardness and oil content of the wastewater sample were found suitable according to the permissible limits of NEQS in Pakistan, whereas BOD and COD values were found to be very high. Similarly, the metal Ni was found in elevated levels as compared to the permissible limit (Table 2).

Wastewater may also be categorized under good and possibly safe categories on the basis of TDS and EC values of 175–525 [16] and 1000–2500 [17], respectively. Bacterial strains identified as *Escherichia coli* and *Bacillus* sp. and fungi identified as *Aspergillus niger* and *Trichoderma pseudokoningii* were detected in the wastewater samples. 

### 2.2. Ni Removal Efficiency of Arthrospira platensis

Ni removal by *Arthrospira platensis* was observed for the synthetic solution, as well as in real effluents with different dilutions (Table 3 and Table 4).

### 2.3. Pollutant Removal Efficiency of Arthrospira platensis

The color of wastewater was reduced to 15 on the index, with an HUE angle of 45.129. Nearly 40.39%, 56.57%, 52.71% and 66.66% decrease was found in BOD, COD, Total Dissolved Solids (TDS) and calcium, respectively, after 16 days of treatment, whereby the concentration of bicarbonates, Mg, Na, K, N and P of samples were also significantly reduced after 16, 33 and 49 days of treatment. (Table 5). The percentage removal of BOD, COD TDS, and calcium was found to be 22.97, 55.07, 36.49 and 33.84 in undiluted wastewater after 16 days of treatment (Table 6).

There were triplicate tanks for the experimental mixture. In cycles 1, 2 and 3, all the values and findings were the same, and there was no significant difference between them after treatment.

There were three replicate tanks for the experimental mixture. In cycles 1, 2 and 3, all the values and findings were the same, and there was no significant difference between them after treatment.

After 6 days, cells aggregated at the bottom of the container and adhesion with the walls was also observed in 100% wastewater. The lag phase was not significant in either experimental mixture. By the sixth week, large masses of bright green filaments had appeared dispersed in the entire container. Such algal biomass can be easily harvested with the help of a net. The growth of *A. platensis* was noted based on dry weight, ash free weight and optical density, which were 41.66 mg L^−1^, 6.66 mg L^−1^, and 2.61, respectively, against the control tank without wastewater.

Desorption was carried out using EDTA_2_Na. The desorption ratio was reduced after each cycle from 50.9 to 29.6 and 28.57, respectively. The biomass data in terms of bioremediation potential are presented in Table 7.

### 2.4. Study of Adsorption Isotherms

**Figure 1:**
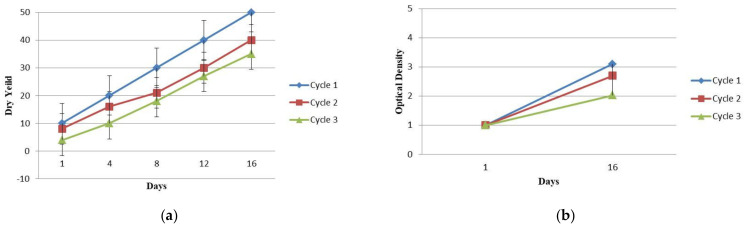
*Arthrospira* biomass growth in aerated tanks after 16, 33 and 49 days of treatment cycle in 100% wastewater. (**a**) Dry yield; (**b**) Optical density.

**Figure 2:**
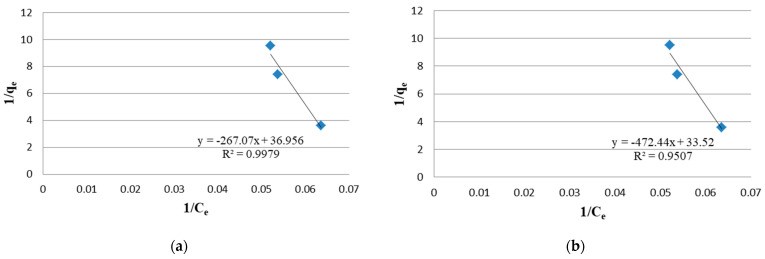
Langmuir Isotherm for the removal of Ni ions from the absorbent. (**a**) In diluted wastewater; (**b**) In undiluted wastewater.

**Figure 3:**
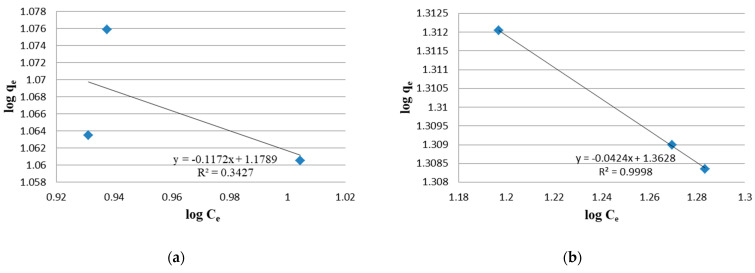
Freundlich Isotherm for the removal of Ni ions from the absorbent. (**a**) In diluted wastewater; (**b**) In undiluted wastewater.

The biomass growth of *Arthrospira* in aerated tanks after 16, 33 and 49 days of treatment cycle in 100% wastewater is plotted in terms of dry yield and optical density (Figure 1a,b). The plots of the linear Langmuir (Figure 2a,b) and Freundlich isotherm models (Figure 3a,b) explain the relationship between the adsorbed amounts of Ni on *Arthrospira platensis* and the concentration remaining in the effluent. Each value is the mean of three replicates. By comparing the R^2^ of the Freundlich model (0.9998) with that obtained from the Langmuir model (0.9507), it was observed that the Freundlich isotherm model best fits the equilibrium data in undiluted wastewater. However, for diluted wastewater, the biosorption equilibrium data was a better fit in the Langmuir model (0.9979) than in the Freundlich isotherm (0.3427).

## 3. Discussion

Microbial bioremediation is the process of removing contaminants or pollutants from wastewater using microbes, such as bacteria, fungi and algae. The contaminants are either removed or transformed into non-hazardous compounds in the water, allowing the water to be reused for irrigation, fish farming and other purposes. In the present study, *Arthrospira* is a widely used microalgae for the remediation of wastewater [18]. Living biomass multiplies continuously, increasing the rate of sequestration of heavy metals selectively and specifically within cells [19].

During the In-Vitro experiment, the biomass rapidly and passively absorbed nickel from the solution. Then, slowly by active metabolism, the metal is transported across the cell membrane. With an increase in pH, metal concentration sorption also increases until equilibrium is attained. An increase in pH in most cases increases solubility, bioavailability and toxicity of heavy metals [20]. Metal adsorption is low at a low pH, probably due to electrostatic repulsion and also because H_3_O^+^ occupies all binding sites. However, with increasing pH, the binding capacity increases directly as more cations and protons are attracted to the negatively charged sites. Sorption at a high pH is only due to the biosorption mechanism, as supported by Iqbal and Saeed [21]. From beginning to end, the pH of the medium in each flask was 7.4–9.2. Less biosorption in dilutions was probably due to the presence of other metal ions in wastewater that compete for the active binding site of the substrate [22]. The indigenous microbiota, on the other hand, enhanced the remediation process in both spiked and dilution sets [23].

Changes in weather conditions lead to variations in temperature, which is a major concern in commercial treatment plants [24]. Therefore, in this study, the tank experiment was conducted in a natural environment to study the potential of *Arthrospira* in this regard, as it has the ability to adsorb trace and toxic metals up to 10% of its biomass [25]. They are capable of doubling their biomass every 24 h and their metal accumulation potential is directly related to metal concentration in aqueous medium. The reduction of organic and inorganic compounds was mainly due to incorporation in biomass, especially in diluted wastewater, while a rise in DO and pH is due to photoheterotrophic metabolism. They form complexes on binding sites with heavy metals and the pollutants of wastewater, leading to flocculation and a reduction in pollution load [26]. The results were parallel to Tripathi, Gupta and Thakur [27], who reported a significant reduction in parameters of wastewater as compared to the control in sterilized wastewater concentrations of 25, 50 and 100% with *Scenedesmus* sp. in BG-11 medium for 2 weeks.

After 15 days, the minerals start to decline, resulting in the death of biomass and a drop in pH to 7.7. This may be explained by the utilization of organic acids and carbonates for photosynthesis. In the initial phase, the growth was slow, increased significantly by the 3rd day and ended with no further changes in biomass until the 15th day. Similar results were reported by Zhou et al. [23]. *A. platensis* continues the production of pigments, regardless of heavy metals [23].

Chemical precipitation and ion exchange is the most employed and established process in heavy metal removal in industries but results in the production of solid sludge in bulk. Biosorption, despite its multiple advantages, is not yet e as the industries find it difficult to adapt novel methods. However, it may be a useful and fruitful, cost-effective approach for developing countries in terms of technology [27]. Regeneration of biomass reduces the cost of wastewater treatment. Desorption at a low pH is favorable and leads to the precipitation of metal hydroxides. Since EDTA is the most common chelating agent used for desorption of metals, its application in the present study was 30% less than the percentage evaluated by Zinicovscaia et al. [28] with HCl, HNO_3_, CH_3_COOH and NaOH. The heavy metals present in wastewater may be recovered from biomass by the process of electrolysis and desorption of the sorbent [29].

Microalgae with a high ash content of around 20% may prove to be good fertilizers. They are used in aquaculture, wastewater treatment, food, fertilizers and maintenance of soil fertility [30] after removal of metals. *Arthrospira* may provide valuable minerals to enhance soil organic carbon, which enhances soil quality and crop growth [31]. The reused and contaminated biomass in this study was discarded from the geomembrane. Wastewater treatment by using microalgae is economical, efficient and environmentally friendly, satisfying both the financial and environmental goals requirements with merely one percent of electricity consumption saving 50–70% of energy and reducing CO_2_ emission along with recycling minerals and clear water. Hence, cyanobacterial systems may be incorporated into primary wastewater treatment plants, as advised by Garbowski et al. [32] and Zinicovscaia et al. [28].

As a significant portion of Ni was desorbed on application of EDTA_2_Na the surface bio adsorbed fraction of Ni would require quantification of bio absorbed fraction with maximum possible empiricism in case of multimetal solution. These findings show that *A. platensis* could potentially be used to remove nickel from nickel-contaminated oil industrial effluents. Clean water and sanitation for all is the sixth sustainable development goal (SDG), which could be achieved by promoting such phycoremediation projects. The isolation and purification of further strains of hyperaccumulator algae from polluted wastewater can be explored in the future, so the treatment spectrum of the mixed wastewater could be expanded. The biochemical mechanism of heavy metal tolerance in the screened hyperaccumulator algae could be examined. Algal may be applied as a metal recovery and recycling tool through the process of algal biomining.

## 4. Materials and Methods

### 4.1. Sample Collection and Pre-Analysis

The grab collection method was adopted at various random points for isolation of green fuzzy microalgae masses from Saim Drain, and also for collection of wastewater from Prime Oil and Ghee Industry (32.03563° N, 74.12432° E). The experimental work was carried out from December 2020 to February 2021 at the University of Punjab, Lahore. Physical parameters, such as appearance, color, odor and turbidity, and chemical parameters, such as pH, EC, temperature, TDS and NaCl, were observed on-site with the help of pH (WTW Series Inolab) and EC meter (HANNA HI 9835) according to APHA [33].

Specimens were separated by centrifugation at 4000 rpm for 10 min and observed under the low and high magnification power of the microscope. Identification was made based on morphological and phenotypic characteristics by an expert according to the literature (Geitler, [34]) based on features such as helix shape, extent and width of coil, cell length and pointed calyptras. *Arthrospira platensis* was selected as the experimental strain. About 5 g of *A. platensis* was isolated from the fuzzy masses by centrifugation. was cultured in flasks containing 50 mL BG-11 medium at 20 ± 5 °C on an orbital shaker, provided with 2000 lux light having 12/12 h light and dark period for a duration of 3 weeks [35]. The biomass was harvested by centrifugation at 4000 rpm for 10 min for analysis of wet weight, dry weight, organic dry weight, chlorophyll content, phycocyanin content, extraction yield, total organic carbon, optical density, total cell count, productivity and growth yield according to Borowitzka and Moheimani [36] and Richmond and Hu [37].

The wastewater sample was subjected to pre-analysis for BOD, COD, DO, TS, TSS, TVS, carbonates, bicarbonates, chlorides, sulfates, Ca, Mg, hardness, Na, K, TKN, total phosphates, oil and Ni according to APHA [33] along with identification of bacterial and fungal community on Lysogeny Broth (LB) and 2% Malt Extract Agar (MEA), respectively. Bacterial identification was made based on morphology, including colony size, shape, surface, color, margin, texture, pigmentation, opacity, elevation, growth, arrangement and motility, whereas fungal colonies were identified on the basis of mycelial morphology observed under microscope.

### 4.2. Batch Experiment

Two batches were prepared in Erlenmeyer flasks. The first batch contained autoclaved dilutions of 10, 20, 30, 40, 50, 60, 70, 80, 90 and 100% wastewater and 2, 4, 6, 8, 10, 12, 14, 16, 18 and 20 ppm solution of NiSO_4_·6H_2_O respectively whereas the second replicate batch was not autoclaved. Both were then inoculated with 2 g of *Arthrospira platensis*, which was separated from the cultivation medium at a growth yield of 0.03. Three replicate flasks were taken for each dilution. The control consisted of *A. platensis*, provided with freshwater and media. The pH of the solutions was adjusted using 0.1 N HCl or NaOH. These were then placed in an orbital incubator at 100 rpm, with a temperature of 25 °C for 3 days. Ni was determined by digestion of the filtrate with Nitric and Perchloric acid (1:1) and analyzed spectrophotometrically by Atomic Absorption Spectrophotometer, AAS (GBC SAVAANT AA Australia) at 232.0 nm. The percentage removal was determined as follows:

(1) $$Q_{e}=\frac{\left(C_{o}-C_{e}\right)}{M}\;V$$

where *Q_e_* is amount of Ni uptake, *C_o_* is initial metal concentration in medium (mg L^−1^), *C_e_* is metal concentration at equilibrium in medium (mg L^−1^), *V* is the solution volume (L) and *M* is the mass of biosorbent (g).

### 4.3. Culture Tank Experiment

To mimic the aeration tank system of primary treatment in wastewater plants with exposure to environmental conditions, two sets were prepared: the first set of diluted wastewater (50% wastewater and 50% freshwater) and the second set of undiluted wastewater (100% wastewater). Each set consisted of four tanks, which included one control (without wastewater) and three replicates of diluted and undiluted wastewater, respectively. Approximately 100 g (wet weight) of *Arthrospira platensis* from the culture was used for inoculation into each of the eight plastic tanks containing 5 L wastewater, pH 9 in a greenhouse. The annual mean temperature from January to March 2021 was 15.55–27.25 ± 5 °C with an average humidity of 70–55% and sunlight intensity of 1850 μmol photons m^2^ s^−1^. Aeration was provided by an SOBO aquarium (SB-348A) air pump. To avoid clumping and settling the cells, the tanks were also manually stirred twice a day. The entire experiment lasted 49 days, which were divided into three 16-day cycles. After each cycle, the physicochemical parameters of the wastewater were analyzed in replicates. The specific growth rate, bioconcentration factor, and mineral uptake of *Arthrospira platensis* were also determined according to Fourooghifard et al. [38], Gupta and Kumar [39] and Dębowski et al. [40], respectively.

Upon harvesting the biomass after an interval of 16 days, the reusability of cyanobacterium was determined by placing filtered biomass collected from the tank into 0.1 M EDTA_2_Na for 2 h at 25 °C, and 100 rpm in an orbital incubator (GLSC OIC CR-196-11). It was then again placed in a tank for another 16 days. The final metal concentration in the solution was determined by AAS after digestion (Gupta, Rastogi and Nayak, [41]). The percentage of desorbed species is estimated as:

(2) $$\mathrm{Desorption}\;\mathrm{ratio}\;\%=\frac{\mathrm{amount}\;\mathrm{of}\;\mathrm{metal}\;\mathrm{ions}\;\mathrm{desorbed}}{\mathrm{amount}\;\mathrm{of}\;\mathrm{metal}\;\mathrm{ions}\;\mathrm{absorbed}}\times 100$$

At the end of the experiment, the biomass was collected and oven dried at 100 °C for 6 h. Then, the ash content was obtained by combustion at 550 °C for 6 h.

### 4.4. Equilibrium Isotherm

Langmuir and Freundlich isotherm models were applied to interpret the results obtained for the removal of Ni through biosorption to describe the equilibrium between metal ions adsorbed on the surface and within the biomass. The metal content remaining in the solution was calculated as the bioconcentration factor and removal percentage [42]. The Langmuir model described the equilibrium between the absorption capacity of adsorbate and the solution affinity of the adsorbent system, whereas the Freundlich model depicted equilibria between the biomass membrane and feed solution.

### 4.5. Statistical Analysis

The average and standard deviation were calculated for each evaluated wastewater parameter. The batch experiment was subjected to one-way ANOVA, using Microsoft Excel 2010, version Windows 2010 with a 0.05% significance level, and the least significant difference test was applied to determine the most suitable concentration for biosorption.

## 5. Conclusions

The Ni biosorption ability of *Arthrospira platensis* Gomont was significantly higher in cooking oil industry effluent than in Ni spiked solution due to the presence of multiple minerals that fulfilled nutrient requirements of the cyanobacterium for growth. Based on the treatment efficiency and survivability of the cyanobacterium in Ni-loaded industrial effluent, it may possibly be taken to the microcosmic level with a probable 50–70% lesser consumption of energy and low carbon footprints. However, the application of the selected cyanobacterium at a larger level would face a huge challenge of keeping its inoculum pure in open ponds during the total time of treatment of a batch of industrial effluent. Since the assessment of the biosorption potential of *Arthrospira platensis* for the treatment of Ni-contaminated wastewater has been considered previously, this research is the first attempt at remediation of effluent from the cooking oil industry by autochthonous *Arthrospira platensis* along with indigenous microbiota under laboratory and natural conditions.

## Figures and Tables

**Table 1:** Biomass assessment of cultured *Arthrospira platensis* in BG-11 medium over a span of 21 days.

Parameter	Value
Wet weight (mg L^−1^)	7972 ± 2.05
Dry weight (mg L^−1^)	1123 ± 3.29
Organic dry weight (mg L^−1^)	775.00 ± 0.94
Biomass productivity	0.81 ± 0.04
Chlorophyll content (mg L^−1^)	8.00 ± 2.9
Phycocyanin content (mg L^−1^)	100.00 ± 2.35
Extraction yield	11.00 ± 0.47
Optical Density	3.01 ± 0.004
Total Cell Count	4.00 ± 0.408
Total Organic Carbon (mg L^−1^)	2.50 ± 0.24
Growth yield	0.03 ± 0.01

mg—milligram, mg L^−1^—milligram per liter.

**Table 2:** Physico-chemical analysis of wastewater from the oil and ghee industry.

Physico-Chemical Parameter	Value	Permissible Limit of NEQS Pakistan (WHO or AEP)
pH	8.21 ± 0.012	6–10
EC (µS/cm)	1020.00 ± 2.9	1000–2500 (AEP)
Temperature (°C)	17.70 ± 0.12	40
TDS (mg L^−1^)	211.00 ± 0.47	3500
NaCl (%)	1.10 ± 0.12	----
BOD (mg L^−1^)	126.90 ± 1.25	80
COD (mg L^−1^)	371.60 ± 0.24	150
DO (mg L^−1^)	0.20 ± 0.09	5 (WHO)
TS (mg L^−1^)	252.00 ± 0.94	----
TSS (mg L^−1^)	41.00 ± 1.24	150
TVS (mg L^−1^)	19.00 ± 0.81	----
CO_3_ (mg L^−1^)	ND	----
HCO_3_ (mg L^−1^)	2.00 ± 0.4	----
Cl (mg L^−1^)	43.78 ± 0.16	1000
S (mg L^−1^)	53.40 ± 0.32	600
Ca (mg L^−1^)	13.00 ± 0.23	75
Mg (mg L^−1^)	3.40 ± 0.05	150
Hardness (mg L^−1^)	46.46 ± 0.008	200
TKN (mg L^−1^)	10.65 ± 0.03	50 (WHO)
TP (mg L^−1^)	1.32 ± 0.01	5 (WHO)
Na (mg L^−1^)	28.00 ± 1.24	----
K (mg L^−1^)	13.00 ± 1.63	----
Oils (mg L^−1^)	8.00 ± 0.81	10
Ni (mg L^−1^)	21.30 ± 0.16	1.0

EC—Electrical conductivity, NaCl—Sodium Chloride, BOD—Biological Oxygen Demand, COD—Chemical Oxygen Demand, TDS—Total Dissolved Solids, TS—Total Solids, TSS—Total Suspended Solids, TVS—Total Volatile Solids, CO_3_—Carbonates, HCO_3_—Bicarbonates, Cl—Chlorides, S—Sulphates, Ca—Calcium, Mg—Magnesium, TKN—Total Kjeldhal Nitrogen, TP—Total Phosphates, Na—Sodium, K—Potassium, Ni—Nickel, ND—not detected.

**Table 3:** Removal of Ni from wastewater batches using *A. platensis* after a period of 3 days.

Wastewater Dilution	Concentration of Ni (mg L^−1^)			Percentage Reduction	
	(Real Effluent)	Batch 1 (without Microbes)	Batch 2 (with Indigenous Microbes)	Batch 1 (without Microbes)	Batch 2 (with Indigenous Microbes)
10%	3.21	2.34 ± 0.22	1.47 ± 0.17	27.04	54.04
20%	5.60	4.27 ± 0.31	3.71 ± 0.26	23.71	33.73
30%	7.13	5.40 ± 0.42	3.60 ± 0.36	24.17	49.5
40%	9.08	5.88 ± 0.41	3.84 ± 0.48	35.24	57.63
50%	11.52	6.26 ± 0.58	5.21 ± 0.27	45.64	54.75
60%	13.74	7.51 ± 0.36	5.52 ± 0.61	45.29	59.81
70%	15.95	7.44 ± 0.48	6.65 ± 0.39	53.32	58.28
80%	17.60	8.91 ± 0.21	6.98 ± 0.53	49.36	60.3
90%	19.84	11.20 ± 0.54	9.46 ± 0.44	43.53	52.27
100%	21.30	18.40 ± 0.72	15.84 ± 0.56	13.61	25.6
LSD		10.09	9.13	-	-

**Table 4:** Removal of Ni from synthetic solution (NiSO_4_·6H_2_O) batches using *A. platensis* after a period of 3 days.

Concentration of Synthetic Solution (mg L^−1^)	Concentration of Ni (mg L^−1^)		Percentage Reduction	
	Batch 1 (without Microbes)	Batch 2 (with Indigenous Microbes)	Batch 1 (without Microbes)	Batch 2 (with Indigenous Microbes)
2	1.79 ± 0.54	1.39 ± 0.53	10.15	30.30
4	3.96 ± 0.47	3.19 ± 0.39	1.00	20.07
6	5.81 ± 0.33	4.40 ± 0.19	3.03	26.66
8	7.13 ± 0.26	6.69 ± 0.74	10.86	16.30
10	9.71 ± 0.68	9.42 ± 0.12	2.87	5.76
12	11.62 ± 0.61	11.12 ± 0.83	3.166	7.33
14	12.05 ± 0.19	12.67 ± 0.47	13.89	9.43
16	15.63 ± 0.77	14.12 ± 0.41	2.30	11.72
18	15.34 ± 0.39	14.16 ± 0.28	14.76	21.31
20	17.62 ± 0.25	17.23 ± 0.55	11.88	13.82
LSD	12.03	12.04	12.16	18.60

**Table 5:** Efficiency of *Arthrospira platensis* in diluted wastewater (50% freshwater and 50% effluent) after different days of treatment for the reduction of pollution load.

Parameter	Days of Treatment				Percentage Removal after Treatment Days		
	0	16	33	49	16	33	49
EC (µS cm^−1^)	780.00	241.66 ± 1.24	241.00 ± 1.63	241.66 ± 2.05	69.01	69.10	69.01
NaCl (%)	0.40	BDL	BDL	BDL	100	100	100
BOD (mg L^−1^)	118.00	70.33 ± 1.24	69.00 ± 0.81	70.00 ± 0.81	40.39	41.52	40.67
COD (mg L^−1^)	502.00	218.00 ± 1.63	205.66 ± 1.24	215.66 ± 1.24	56.57	59.03	57.04
TDS (mg L^−1^)	129.00	61.00 ± 0.81	62.00 ± 0.81	60.00 ± 0.81	52.71	51.93	53.48
TS (mg L^−1^)	117.00	59.00 ± 1.63	59.00 ± 0.81	57.66 ± 1.24	49.57	49.57	50.71
TSS (mg L^−1^)	12.00	2.66 ± 0.47	3.33 ± 0.47	2.66 ± 0.47	77.77	72.22	77.77
TVS (mg L^−1^)	4.00	0.70 ± 0.244	0.86 ± 0.32	0.93 ± 0.20	82.50	78.33	76.66
CO_3_ (mg L^−1^)	BDL	BDL	BDL	BDL	---	---	---
HCO_3_ (mg L^−1^)	BDL	BDL	BDL	BDL	---	---	---
Cl (mg L^−1^)	20.20	6.50 ± 0.40	6.50 ± 1.08	5.66 ± 1.24	67.82	67.82	71.94
S (mg L^−1^)	31.00	12.66 ± 1.69	11.33 ± 1.24	11.00 ± 0.40	59.13	63.44	64.51
Ca (mg L^−1^)	9.00	3.00 ± 0.81	3.33 ± 0.47	4.00 ± 0.81	66.66	62.96	55.55
Mg (mg L^−1^)	2.10	BDL	0.433 ± 0.12	0.33 ± 0.12	100	79.365	84.12
Hardness (mg L^−1^)	35.17	11.56 ± 0.04	11.41 ± 0.17	11.27 ± 0.03	67.11	67.55	67.93
TKN (mg L^−1^)	5.07	0.11 ± 0.04	0.12 ± 0.01	0.19 ± 0.02	97.83	97.63	96.25
TP (mg L^−1^)	0.20	BDL	BDL	BDL	100	100	100
Na (mg L^−1^)	12.00	3.81 ± 0.41	4.03 ± 0.19	4.16 ± 0.32	68.25	66.41	65.33
K (mg L^−1^)	6.00	0.52 ± 0.07	0.54 ± 0.03	0.59 ± 0.06	91.33	91	90.16
Oils (mg L^−1^)	5.00	2.93 ± 0.73	2.93 ± 0.68	2.93 ± 0.71	41.40	41.40	41.40
Ni (mg L^−1^)	12.00	8.53 ± 2.16	8.66 ± 1.03	10.1 ± 0.86	28.88	27.77	15.83

EC—Electrical conductivity, NaCl—Sodium Chloride, BOD—Biological Oxygen Demand, COD—Chemical Oxygen Demand, TDS—Total Dissolved Solids, TS—Total Solids, TSS—Total Suspended Solids, TVS—Total Volatile Solids, CO_3_—Carbonates, HCO_3_—Bicarbonates, Cl—Chlorides, S—Sulphates, Ca—Calcium, Mg—Magnesium, TKN—Total Kjeldhal Nitrogen, TP—Total Phosphates, Na—Sodium, K—Potassium, Ni—Nickel, BDL—Below Detection Limit.

**Table 6:** Efficiency of *Arthrospira platensis* in undiluted wastewater after different days of treatment for the reduction of pollution load.

Parameter	Days of Treatment				Percentage Removal after Treatment Days		
	0	16	33	49	16	33	49
EC (µS cm^−1^)	1020.00	586.66 ± 2.05	565.00 ± 1.41	569.00 ± 0.81	42.48	44.60	44.21
NaCl (%)	1.10	0.80 ± 1.11	0.80 ± 1.11	0.80 ± 1.11	27.27	27.27	27.27
BOD (mg L^−1^)	162.90	125.46 ± 2.73	132.00 ± 1.20	128.73 ± 0.83	22.97	18.96	20.97
COD (mg L^−1^)	751.60	337.66 ± 1.24	372.66 ± 2.05	380.33 ± 1.24	55.07	50.42	49.40
TDS (mg L^−1^)	211.00	134.00 ± 3.26	122.33 ± 2.05	119.66 ± 1.24	36.49	42.02	43.28
TS (mg L^−1^)	252.00	194.66 ± 1.24	186.00 ± 2.16	183.66 ± 1.24	22.75	26.19	27.11
TSS (mg L^−1^)	41.00	31.33 ± 1.24	28.00 ± 1.63	26.33 ± 1.24	23.57	31.70	35.77
TVS (mg L^−1^)	19.00	11.66 ± 1.24	10.00 ± 0.81	9.33 ± 1.24	38.59	47.36	50.87
CO_3_ (mg L^−1^)	0	---	---	---	---	---	---
HCO_3_(mg L^−1^)	2.00	BDL	BDL	BDL	100	100	100
Cl (mg L^−1^)	43.78	15.00 ± 0.81	16.66 ± 0.94	15.66 ± 1.24	65.73	61.93	64.21
S (mg L^−1^)	53.40	22.66 ± 1.69	20.00 ± 0.81	18.76 ± 1.11	57.55	62.54	64.85
Ca (mg L^−1^)	13.00	8.60 ± 0.43	8.00 ± 0.81	9.00 ± 0.81	33.84	38.46	30.76
Mg (mg L^−1^)	3.40	BDL	0.11 ± 0.03	0.24 ± 0.06	100	96.76	92.94
Hardness (mg L^−1^)	46.46	26.36 ± 0.45	24.03 ± 0.82	25.23 ± 1.06	43.25	48.26	45.69
TKN (mg L^−1^)	10.65	1.42 ± 0.26	1.64 ± 0.31	2.10 ± 0.22	86.66	84.60	80.28
TP (mg L^−1^)	1.32	BDL	BDL	BDL	100	100	100
Na (mg L^−1^)	28.00	7.24 ± 0.62	7.64 ± 0.18	8.10 ± 0.28	74.14	72.71	64.78
K (mg L^−1^)	13.00	4.12 ± 0.75	4.46 ± 0.81	4.99 ± 0.79	68.30	65.69	61.61
Oils (mg L^−1^)	8.00	3.00 ± 0.81	5.33 ± 0.47	4.33 ± 1.24	62.5	33.33	45.83
Ni (mg L^−1^)	21.30	15.73 ± 0.46	18.60 ± 0.74	19.20 ± 0.24	26.13	12.67	9.85

EC—Electrical conductivity, NaCl—Sodium Chloride, BOD—Biological Oxygen Demand, COD—Chemical Oxygen Demand, TDS—Total Dissolved Solids, TS—Total Solids, TSS—Total Suspended Solids, TVS—Total Volatile Solids, CO_3_—Carbonates, HCO_3_—Bicarbonates, Cl—Chlorides, S—Sulphates, Ca—Calcium, Mg—Magnesium, TKN—Total Kjeldhal Nitrogen, TP—Total Phosphates, Na—Sodium, K—Potassium, Ni—Nickel, BDL—Below Detection Limit.

**Table 7:** Bioremediation potential of *Arthrospira platensis* in 100% wastewater (without dilutions).

Parameter Observed	Value
Specific Growth rate (mg L^−1^)	293.00 ± 136.35
Biomass productivity (mg L^−1^)	133.78 ± 41.98
Mineral uptake	14.24 ± 4.47
Nickel percentage removal	16.22 ± 7.11
Bioconcentration factor	0.11 ± 0.04
Desorption ratio	36.35 ± 10.29

## Data Availability

Not applicable.

## Sample Availability

Samples of the compounds are not available from authors.

## References

[1] Ge S., Madill M., Champagne P. (2018). Use of freshwater macroalgae *Spirogyra* sp. for the treatment of municipal wastewaters and biomass production for biofuel applications. Biomass. Bioenerg..

[2] Elleuch J., Amor F.B., Chaaben Z., Frikha F., Michaud P., Fendri I., Abdelkafi S. (2021). Zinc biosorption by *Dunaliella* sp. AL-1: Mechanism and effects on cell metabolism. Sci. Total Environ..

[3] Ubando A.T., Africa A.D.M., Maniquiz-Redillas M.C., Culaba A.B., Chen W.H., Chang J.S. (2021). Microalgal biosorption of heavy metals: A comprehensive bibliometric review. J. Hazard. Mater..

[4] Gillette B. (2008). Nickel named «Allergen of the Year». ACDS adds to list of substances warranting more attention. Dermatol. Times.

[5] Murtaza G., Zia M.H. (2012). Wastewater Production, Treatment and Use in Pakistan. Second Regional Workshop of the Project ‘Safe Use of Wastewater in Agriculture.

[6] Hussain R., Ahmad W., Nafees M., Hussain A. (2014). Optimization of wastewater treatment process in industry “a case study of Hattar Industrial Estate Haripur”. Pak. J. Anal. Environ. Chem..

[7] El-Gawad A. (2014). Oil and grease removal from industrial wastewater using new utility approach. Adv. Environ. Chem..

[8] Yang Q.Z., Qi G.J., Low H.C., Song B. (2011). Sustainable recovery of nickel from spent hydrogenation catalyst: Economics, emissions and wastes assessment. J. Clean. Prod..

[9] Shabir A. (2016). Process Line of Cooking Oil and Vegetable Ghee (Vanaspati) and their Analysis during Processing. Res. Rev. J. Food Dairy Technol..

[10] Qin H., Hu T., Zhai Y., Lu N., Aliyeva J. (2020). The improved methods of heavy metals removal by biosorbents: A review. Environ. Pollut..

[11] Aketo T., Hoshikawa Y., Nojima D., Yabu Y., Maeda Y., Yoshino T., Takano H., Tanaka T. (2020). Selection and characterization of microalgae with potential for nutrient removal from municipal wastewater and simultaneous lipid production. J. Biosci. Bioeng..

[12] Masojídek J., Torzillo G. (2014). Mass Cultivation of Freshwater Microalgae.

[13] Sili C., Torzillo G., Vonshak A. (2012). *Arthrospira* (*Spirulina*). Ecology of Cyanobacteria II.

[14] Greshwin M.E., Belay A. (2008). Spirulina (Arthrospira): Production and Quality Assurance. Spirulina in Human Nutrition and Health.

[15] Novoa S., Wernand M., van der Woerd H.J. The Forel-Ule scale converted to modern tools for participatory water quality monitoring. Proceedings of the Extended Abstract Ocean Optics Conference XII.

[16] Fipps G. (2004). Irrigation Water Quality Standards and Salinity Management.

[17] AEP (1996). Alberta User Guide for Waste Managers.

[18] Olguín E.J., Galicia S., Mercado G., Pérez T. (2003). Annual productivity of *Spirulina* (*Arthrospira*) and nutrient removal in a pig wastewater recycling process under tropical conditions. J. Appl. Phycol..

[19] Joo G., Lee W., Choi Y. (2011). Heavy metal adsorption capacity of powdered *Chlorella vulgaris* biosorbent: Effect of chemical modification and growth media. Environ. Sci. Pollut. Res..

[20] Weisse T., Stadler P. (2006). Effect of pH on growth, cell volume, and production of freshwater ciliates, and implications for their distribution. Limnol. Oceanogr..

[21] Iqbal M., Saeed A. (2007). Production of an immobilized hybrid biosorbent for the sorption of Ni (II) from aqueous solution. Process Biochem..

[22] Mehta S.K., Gaur J.P. (2005). Use of algae for removing heavy metal ions from wastewater: Progress and prospects. Crit. Rev. Biotechnol..

[23] Zhou J., Wen Y., Wu Y., Wu Y. Notice of Retraction: Effect of Nitrogen and Phosphorus Ratio on Algal Growth in Lake Xuanwu. Proceedings of the 5th International Conference on Bioinformatics and Biomedical Engineering IEEE.

[24] Lawton R.J., de Nys R., Skinner S., Paul N.A. (2014). Isolation and identification of *Oedogonium* species and strains for biomass applications. PLoS ONE.

[25] Rajamani S., Siripornadulsil S., Falcao V., Torres M., Colepicolo P., Sayre R. (2007). Phycoremediation of heavy metals using transgenic microalgae. Transgenic Microalgae as Green Cell Factories.

[26] Balaji S., Kalaivani T., Shalini M., Gopalakrishnan M., Rashith M.M.A., Rajasekaran C. (2016). Sorption sites of microalgae possess metal binding ability towards Cr (VI) from tannery effluents—A kinetic and characterization study. Desalin. Water Treat..

[27] Tripathi R., Gupta A., Thakur I.S. (2019). An integrated approach for phycoremediation of wastewater and sustainable biodiesel production by green microalgae, *Scenedesmus* sp. ISTGA1. Renew. Energy.

[28] Zinicovscaia I., Yushin N., Gundorina S., Demčák Š., Frontasyeva M., Kamanina I. (2018). Biosorption of nickel from model solutions and electroplating industrial effluent using cyanobacterium *Arthrospira platensis*. Desalin. Water Treat..

[29] Gahlout M., Prajapati H., Chauhan P., Savande L., Yadav P. (2017). Isolation, screening and identification of cyanobacteria and its uses in bioremediation of industrial effluents and chromium sorption. Int. J. Adv. Res. Biol. Sci..

[30] Murtaza G., Ghafoor A., Qadir M., Owens G., Aziz M.A., Zia M.H. (2010). Disposal and use of sewage on agricultural lands in Pakistan: A review. Pedosphere.

[31] Emparan Q., Harun R., Danquah M.K. (2019). Role of phycoremediation for nutrient removal from wastewaters: A review. Appl. Ecol. Environ. Res..

[32] Garbowski T., Bawiec A., Pulikowski K., Wiercik P. (2017). Algae proliferation on substrates immersed in biologically treated sewage. J. Ecol. Eng..

[33] Clesseri L.S., Greenberg A.E., Eaton A.D. (1998). Standard Methods for the Examination of Water and Wastewater.

[34] Geitler L. (1932). Cyanophyceae. Kryptogramenflora von Deutschland, Osterreich und der Schweiz.

[35] Prasad R.N., Sanghamitra K., Antonia G.M., Juan G.V., Benjamin R.G., Luis I.M.J., Guillermo V.V. (2013). Isolation, identification and germplasm preservation of different native *Spirulina* species from Western Mexico. Am. J. Plant Sci..

[36] Borowitzka M.A., Moheimani N.R. (2013). Algae for Biofuels and Energy.

[37] Richmond A., Hu Q. (2013). Handbook of Microalgal Culture.

[38] Fourooghifard H., Matinfar A., Mortazavi M.S., Roohani G.K., Roohani G.M. (2018). Nitrogen and phosphorous budgets for integrated culture of whiteleg shrimp *Litopenaeus vannamei* with red seaweed *Gracilaria corticata* in zero water exchange system. Iran. J. Fish. Sci..

[39] Gupta S., Kumar A. (2019). Removal of nickel (II) from aqueous solution by biosorption on *A. barbadensis* Miller waste leaves powder. Appl. Water Sci..

[40] Dębowski M., Rusanowska P., Zieliński M., Dudek M., Romanowska-Duda Z. (2018). Biomass production and nutrient removal by *Chlorella vulgaris* from anaerobic digestion effluents. Energies.

[41] Gupta V.K., Rastogi A., Nayak A. (2009). Biosorption of nickel onto treated alga (*Oedogonium hatei*): Application of isotherm and kinetic models. J. Colloid. Interf. Sci..

[42] Al-Homaidan A.A., Al-Abbad A.F., Al-Hazzani A.A., Al-Ghanayem A.A., Alabdullatif J.A. (2016). Lead removal by *Spirulina platensis* biomass. Int. J. Phytoremediation.

